# Comparison of sensitivity of rhesus and cynomolgus macaque for acute radiation effects

**DOI:** 10.1038/s41598-025-10094-y

**Published:** 2025-07-08

**Authors:** Vijay K. Singh, Issa. Melendez-Miranda, Oluseyi O. Fatanmi, Stephen Y. Wise, Alana D. Carpenter, Sarah A. Petrus, Luis A. Lugo-Roman, Thomas M. Seed

**Affiliations:** 1 Division of Radioprotectants, Department of Pharmacology and Molecular Therapeutics, F. Edward Hébert School of Medicine, Uniformed Services University of the Health Sciences, 4301 Jones Bridge Road, Bethesda, MD 20814 USA; 2 Armed Forces Radiobiology Research Institute, Uniformed Services University of the Health Sciences, Bethesda, MD 20814 USA; 3 Department of Laboratory Animal Resources, Uniformed Services University of the Health Sciences, Bethesda, MD 20814 USA; 4 Tech Micro Services, 4417 Maple Avenue, Bethesda, MD 20814 USA

**Keywords:** Acute radiation syndrome, Blood cell counts, Blood chemistry, Cynomolgus, Cytokines, Histopathology, Nonhuman primates, Radiation dose, Rhesus, Survival, Biomarkers, Experimental models of disease

## Abstract

Radiation medical countermeasure development under the United States Food and Drug Administration Animal Rule needs validated large animal models of acute radiation syndrome. Such a well-established large animal model is the rhesus nonhuman primate. The potential use of the rhesus for other high priority areas and limited supply of such animals emphasizes the need to validate other large animal models, in particular other macaque models, in order to compensate for the lack of rhesus macaques for radiation countermeasure development. Based on existing data, cynomolgus macaques, *Macaca fascicularis*, are a viable alternative, but need further characterization. Reliance on such animal models requires that the models are well validated. Data gathered from rhesus and cynomolgus macaques under the same experimental conditions are not available; therefore, the authors compared and contrasted here the radiosensitivity of both macaques receiving same levels of clinical support and exposed to same doses of total-body gamma-radiation, and at same dose rates using same radiation source. Under matched experimental conditions, significant differences between acutely irradiated rhesus and cynomolgus macaques relative to the rates of survival and blood cell changes were observed. The presented data demonstrate that the cynomolgus macaque is more sensitive to ionizing radiation exposure. Overall, data supports the concept that the cynomolgus macaque is a viable, potentially useful alternative large animal model for the evaluation of radiation medical countermeasures. For comparative purposes however, additional studies with both macaques under identical experimental conditions, such as levels of clinical support and different radiation qualities with males and females ran concurrently in future will be critically important.

**Supplementary Information:**

The online version contains supplementary material available at 10.1038/s41598-025-10094-y.

## Introduction

Exposure to ionizing radiation is an undisputable reality of life, and as such, can carry an array of uncertain health risks, some of which can be both serious and consequential^1^. Radiological/nuclear preparedness and availability of radiation medical countermeasures (MCMs) are serious security issues for the individual and the Nation^2^. Radiation exposures can result in different types of injuries, requiring diagnostic and therapeutic measures for treatment. The clinical progression of acute radiation syndrome (ARS) depends on the acuteness of the exposure magnitude^3^. The extent of radiation-induced injury to hematopoietic, gastrointestinal (GI), and neurovascular systems, lung, skin and other organs/systems plays an important role in the diagnosis, treatment, and prognosis of exposed victims. Since hematopoietic tissues of all mammals are highly sensitive to ionizing radiation, effective diagnostic and therapeutic measures for hematopoietic ARS (H-ARS) are critical to the health and well-being of exposed victims^4^. ARS manifests following total- or partial-body irradiation in humans at estimated radiation doses above 1 Gy and delivered at relatively high dose rates (~ 0.05 Gy/h or higher). Clinical manifestations of three subsyndromes occur with specific dose ranges; hematopoietic ARS (H-ARS, 1–6 Gy), gastrointestinal (GI-ARS, > 6 Gy), and neurovascular (NV-ARS, 10 Gy)^5^.

Development and availability of safe and effective MCMs are deemed essential in order to protect individuals and the public at large following unwanted high dose radiation exposures. Deficits in these areas currently reflect a significant unmet medical need and, as such, are considered as a high priority area^6^. Importantly, having the comprehensive knowledge of the strengths and weaknesses of given experimental model systems are of paramount importance for MCM development^7–9^. Due to the ethical constrains of irradiating and testing MCMs in human volunteers, the use of appropriate, well-investigated large animal models for MCM investigation are an integral and essential component to the MCMs-Research and Development process. Accordingly, the United States Food and Drug Administration (US FDA) has guidance, commonly known as the Animal Rule, for regulatory approval of such agents needed to thwart chemical, biological, radiological, and nuclear threats^10–12^.

Irradiation-based injury models that employ nonhuman primates (NHPs) may generate data suitable for comparison to the treatment provided to radiation accident victims^13–18^. Due to these positive attributes, models of total- and partial-body irradiation (TBI and PBI) have been developed in NHPs, which play an important role in the development of FDA-approved MCMs^19–28^.

The rhesus macaque, *Macaca mulatta*, has been the predominant NHP model for assessing the biology of several cytokines/growth factors efficacy of various promising MCMs for ARS^29,30^. The cynomolgus macaque, *Macaca fascicularis*, has been used to a lesser extent but this model is no less effective in defining cytokine biology in healthy animals and subsequent use for investigating various MCMs^31–33^.

Thus, the cynomolgus macaque needs to be characterized and compared with rhesus to validate it as a suitable animal model for the development of MCMs following the US FDA Animal Rule. In this study with a limited number of rhesus and cynomolgus macaques and two different radiation doses, we have compared these two macaques under identical conditions to judge the suitability of a large animal model for use in the development of MCMs. Our study demonstrates that these two macaque models of TBI are similar in general terms, although cynomolgus appears more sensitive to ionizing radiation exposure. Furthermore, their complete blood counts (CBC) recovery profiles appear to be somewhat different.

## Materials and methods

### General experimental design

Rhesus (*M. mulatta*) and cynomolgus (*M. fascicularis*) comparisons of lethally irradiated macaques were made using male animals. These animals were exposed to two different doses, 6.5 and 5.8 Gy, of total-body gamma-radiation using a high-level cobalt-60 source to induce acute injury to vital organ systems^18,34,35^. The 5.8 and 6.5 Gy radiation doses are LD_30/60_ and LD_50/60_, respectively, for rhesus. The same doses were used for cynomolgus for comparison though lethality of these doses is higher in cynomolgus. There were 8 cynomolgus macaques in each radiation group; whereas for the rhesus macaques, there were 10 NHPs used in each of the radiation groups. Survival and hematopoietic recovery based on CBC analysis, in addition to other associated parameters, were used for comparison of these two macaques. The study was conducted for 60 days post-irradiation.

### Animals

A total of 36 naïve male macaques (twenty *M. mulatta* (Chinese) and sixteen *M. fascicularis* (Mauritius), were used for the study presented here. The animals’ ages ranged between 3 and 6 years (*M. mulatta* 3–6 years, *M. fascicularis,* 4–6 years), weighing 4.3–9.0 kg (*M. mulatta* 4.3–9.0 kg, *M. fascicularis* 5.5–7.5 kg). Rhesus macaques were procured from the National Institutes of Health Animal Center (NIHAC, Poolesville, MD, USA) while cynomolgus macaques were supplied by Alpha Genesis Inc. (Yemassee, SC, USA). Procured animals tested negative for Herpes B virus (aka Macacine herpesvirus 1), Simian T-cell leukemia virus type 1 (STLV-1), Simian Immunodeficiency virus (SIV) and Simian Retrovirus (SRV) Types 1, 2, 3, and 5. NHPs were either vaccinated for measles or, in the case of previously measles-vaccinated NHPs, tested for the presence of measles antibodies. In addition, animals came from the vendor colony negative for Salmonella, Shigella, Klebsiella, Yersinia, and Simian varicella virus (SVV). All animal health records were reviewed and selections of animals were made by the attending veterinarian. All animals were maintained in a facility accredited by the AAALAC International organization. Animals were quarantined for six weeks prior to the initiation of the experiments. Animal housing, health monitoring, care, and enrichment during the experimental period have been described in detail earlier^35,36^. The study received approvals from the Institutional Animal Care and Use Committee of Armed Forces Radiobiology Research Institute, Uniformed Services University of the Health Sciences (P-2010-12-017, P-2017-07-008, PHA-22-086) and the Department of Defense Animal Care and Use Review Office (ACURO). All studies were carried out in strict accordance with the recommendations made in the Guide for the Care and Use of Laboratory Animals^37^. This study is reported in accordance with ARRIVE (Animal Research: Reporting of In Vivo Experiments) guidelines.

### Total-body irradiation

All irradiation procedures and dosimetry were reported previously in detail^15^. In brief, food was withheld for 12–18 h prior to irradiation; animals were sedated 30–45 min prior to exposure with intramuscular (*im*) ketamine (10–15 mg/kg) and then placed in separate Plexiglas irradiation boxes. Two NHPs were placed on the irradiation platform and secured in seated, back-to-back positions and exposed to a specific radiation dose of ^60^Co γ-radiation at a dose rate of 0.6 Gy/min on both sides of the abdominal core (bilateral, simultaneous exposure). All irradiation procedures and dosimetry of rhesus were reported and discussed in detail earlier^16^. The radiation field in the area of the NHP location was uniform within ± 1.5%. For rhesus, the dosimetry for photons was based on the alanine/EPR (electron paramagnetic resonance) dosimetry system as described earlier^38^. The dosimetry was calibrated in terms of absorbed dose to water using the US National Standard Radiation Sources. For cynomolgus macaque dosimetry, two polymethylmethacrylate boxes were placed on the exposure table with identical phantoms on foam supports. The phantoms were water-filled cylinders, 34.5 cm long, outer diameters 5.08, 6.99, 10.0, 12.5 and 15.1 cm; the diameter of the sleeve matched the diameter of the ion chamber. Two N30013 ion chambers, manufactured by PTW dosimetry (Freiburg, Germany), were used to measure dose rate as described in American Association of Physicists in Medicine Protocol TG-51^39^. Ion chambers and electrometers were calibrated at the NIST traceable Accredited Dosimetry Calibration Laboratory of University of Wisconsin as required by TG-51. Dose rate in each phantom was measured 4 times with relative standard deviation less than 0.4%. Two different doses of radiation, 5.8 and 6.5 Gy, were used to irradiate animals^38^. The targeted dose rate was 0.6 Gy/min, the actual dose rates for 5.8 and 6.5 Gy were 0.58–0.59 Gy/min. During the irradiation procedure, the NHPs were monitored continuously by closed circuit security cameras. For this study, animals underwent bilateral simultaneous irradiation. At the conclusion of the irradiation, NHPs were transported back to the vivarium and placed in their respective home cage. The NHPs were closely monitored until recovered from the sedative and were able to move around. The rhesus studies were completed between 2015 and 2018 and the cynomolgus studies were conducted in 2024.

### Cage-side animal observations

All NHPs were observed pre-irradiation and for 60 d post-irradiation with survival being the primary measured endpoint. Daily observations for signs of pain and distress were made no less than twice a day by husbandry or research staff. Between days 10 and 20 post-irradiation, animals were observed three times a day approximately 8–10 h apart. All procedures and routine protocols on animal observations have been reported earlier^40^.

### Blood collection

Blood draws were accomplished by venipuncture from the saphenous or brachial veins approximately 1–3 h after animals were fed, and were subsequently analyzed for complete blood counts (CBC). On any given day no more than 1% of total blood volume (calculated based on the body weight of individual animal) was collected These procedures are described extensively in an earlier publication^41^. Similarly, the methodology for serum collection for blood biochemistry and cytokine analysis is described elsewhere^42^. In brief, the blood was placed in serum separating tubes and allowed to clot for at least 30 min prior to being centrifuged (10 min, 400 × g). The separated serum samples were aliquoted and stored at −70 °C until use for specific test.

### CBC analysis

Whole blood samples were counted using a Bayer Advia-120 hematology analyzer (Siemens Medical Solutions, Malvern, PA)^43^ and twenty blood parameters were analyzed: white blood cells (WBC), red blood cells (RBC), hematocrit (HCT), hemoglobin (HGB), mean corpuscular volume (MCV), mean corpuscular hemoglobin (MCH), mean corpuscular hemoglobin concentration (MCHC), platelets, and absolute counts and percentages for neutrophils, lymphocytes, monocytes, eosinophils, basophils, and reticulocytes^14^.

### Blood biochemistry analysis

Serum samples separated from whole blood were analyzed for select, biochemical parameters using a Vitros 350 automatic biochemistry analyzer (Ortho Clinical Diagnostics, Markham, ON, Canada)^18^. The parameters analyzed were as follows: alanine transaminase (ALT), albumin, alkaline phosphatase (ALKP), amylase, aspartate aminotransferase (AST), blood urea nitrogen (BUN), calcium, carbon dioxide, chloride, cholesterol, creatine kinase (CK), creatinine, direct high-density lipoprotein (dHDL), gamma-glutamyl transferase (GGT), glucose, lactate dehydrogenase (LDH), lipase, phosphorus, potassium, sodium, triglycerides, total bilirubin (TB), total protein (TP), and uric acid. The following parameters can be seen in Figs. 5 and 6: sodium, potassium, chloride, ALKP, LDH, AST, ALT, GGT, glucose, creatinine, TP, albumin, amylase, lipase, and uric acid. The other parameters did not show significant differences between the two species.

### Multiplex analysis of cytokines

A Luminex 200 analyzer (Luminex Corp, Austin, TX, USA) was used to measure the cytokine, chemokine, and growth factor concentration in the serum samples using custom-made multiplex kits (Bio-Rad Laboratories, Hercules, CA, USA). A list of the 48 targeted and assessed cytokines has been provided earlier^44^. Standard curves for each cytokine were prepared by serial dilution and run in duplicates. Cytokine concentration (pg/ml) was determined by fluorescence intensity and its quantification was performed using Bio-Plex Manager software, version 6.1 (Bio-Rad Inc.)^45^.

### Medical management/symptomatic palliative care

After specialized procedures, NHPs were monitored at least twice daily for signs of complications. The type of supportive care provided was based on CBC analysis and cage side observations^34,46^. Antibiotics were initiated when the absolute neutrophil count was < 500 cells/µl and continued until the count reached > 500 cells/µl. The primary antibiotic used to prevent potential secondary infections was enrofloxacin (Baytril Bayer HealthCare LLC, Shawnee Mission, KS) and the administered dose was 10 mg/kg administered *im* once daily (QD). Additional supportive care measures included rehydration fluids, alternate antibiotics, antipyretics, antidiarrheal agents, analgesics, antiemetics, treatment for mucosal ulcers, and nutritional support. Blood product was not used in the studies reported here.

#### Euthanasia

Euthanasia was accomplished following the *Guide*, the approved IACUC protocol, and the American Veterinary Medical Association Guidelines for the Euthanasia of Animals^37,47^. When an animal reached a state of moribundity/no return, the animal was euthanized. Moribundity was used as a surrogate for mortality, and euthanasia conducted to minimize pain and distress^48,49^. Considerations for moribundity included weight loss, inappetence, ability to eat or drink, responsiveness to stimuli, body temperature, and severe anemia and thrombocytopenia. Organ system (respiratory, gastrointestinal, urogenital, nervous system, and integumentary) dysfunction was also evaluated by a veterinarian as a consideration for moribundity as well. Animals were monitored a minimum of two times a day after irradiation. Monitoring increased to three times a day during days 11–20 post-irradiation, the ‘critical period’, in which moribundity was most expected. During this period, animals were evaluated at least every 8–10 h. No specific combination of symptoms resulted in a decision of moribundity without the consultation of a veterinarian. All decisions for moribundity, and therefore euthanasia, were determined by a team effort between the veterinarian and veterinary staff, principal investigator and study staff, and husbandry staff. Necropsy was performed on all decedents. The moribund animals were given pentobarbital sodium *iv* (Virbac AH Inc., Fort Worth, TX) using either saphenous or cephalic veins, 100 mg/kg, 1–5 ml). Prior to pentobarbital administration, NHPs were sedated using ketamine hydrochloride injection (Mylan Institutional LLC, Rockford, IL, 5–15 mg/kg, *im*). Intra-cardiac administration was used if unable to administer pentobarbital sodium through peripheral veins. For this purpose, animals were deeply anesthetized by Isoflurane (Baxter Healthcare Corporation, Deerfield, IL, 1–5%) with oxygen at 1–4 L per minute via mask before administering the intra-cardiac injection.

#### Data analysis

Statistical software SPSS v.28 was used for all statistical analyses. For cytokines, CBC, blood chemistry, and survival data, each group was required to have a minimum of three animals in order to determine significance at any given time point. For any comparison with less than three animals in either group, statistical analysis was not performed. All error bars represent standard deviations. Analysis of variance (ANOVA) was used to detect significant differences between rhesus and cynomolgus. The Tukey–Kramer test was used to determine which pairwise comparisons were significant, with the *p* value set at 0.05. For survival data, Chi-square tests were performed to compare survival rates of rhesus and cynomolgus.

## Results

### Survival study

**Fig. 1:**
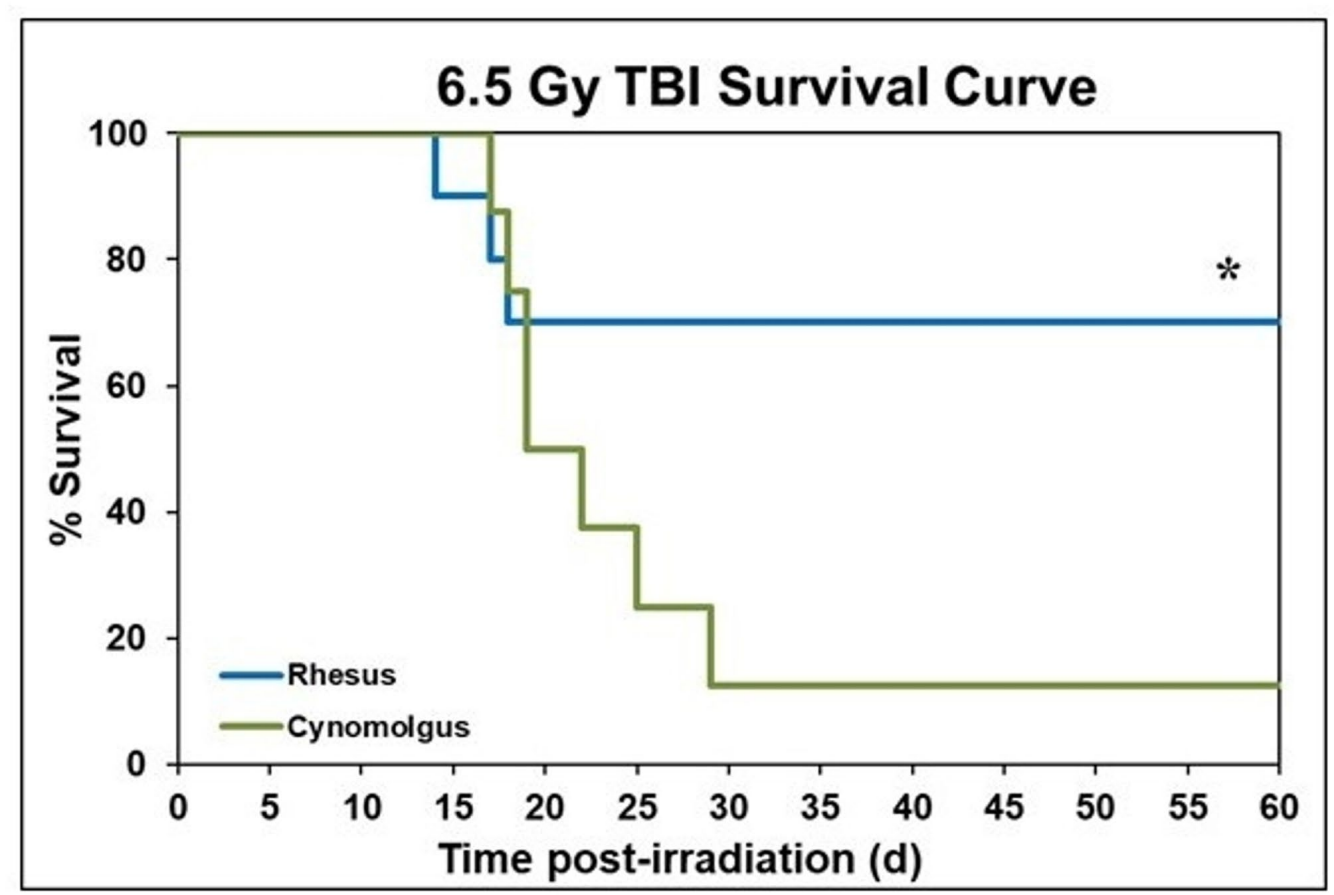
Kaplan-Meyer survival curve demonstrating the 60-day survival of male Rhesus and Cynomolgus macaques exposed to 6.5 Gy total-body gamma radiation. A chi-square test was performed with SPSS to detect significant differences between species. An * indicates statistical significance (*p* < 0.05).

**Fig. 2:**
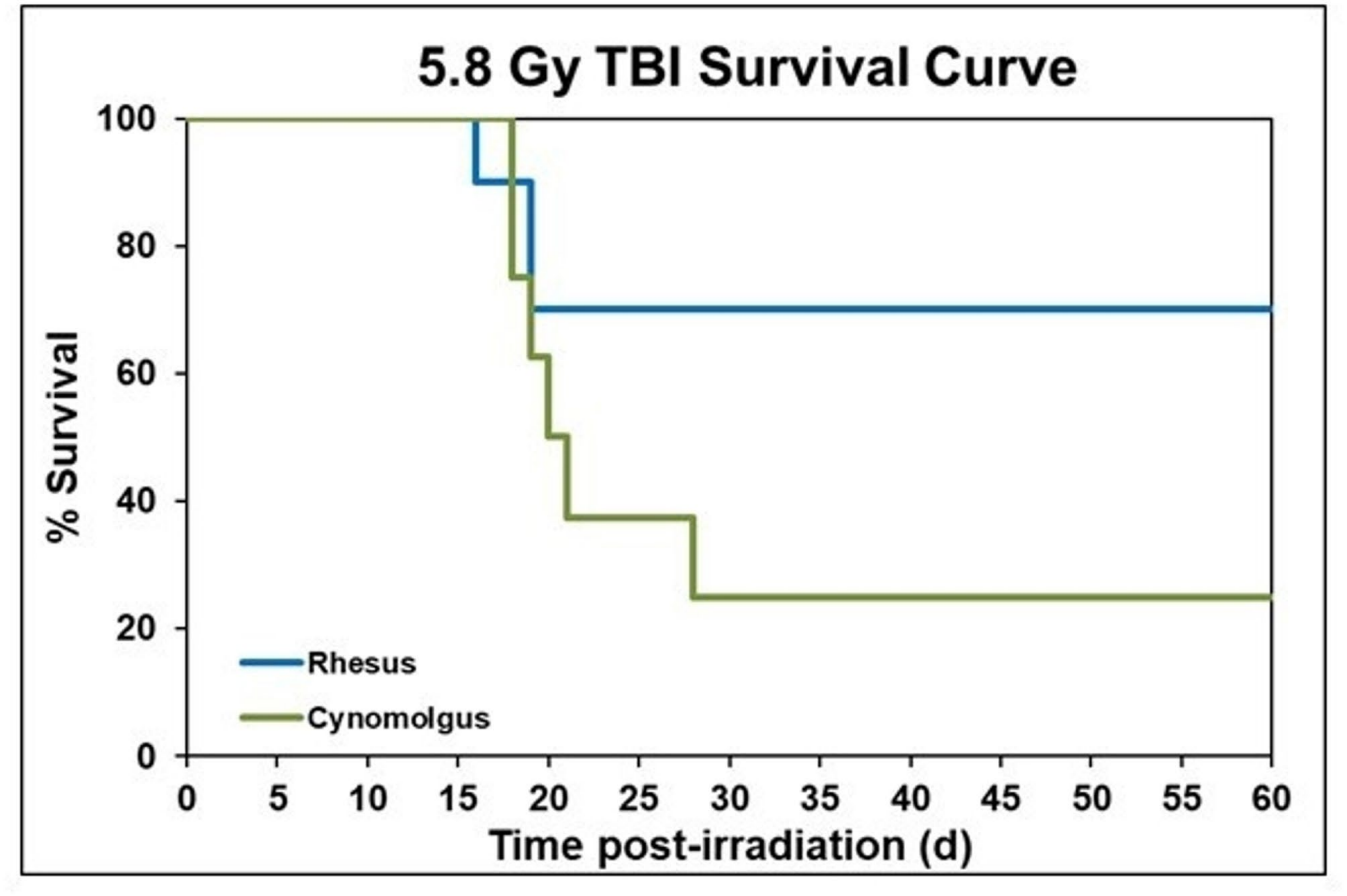
Kaplan-Meyer survival curve demonstrating the 60-day survival of male Rhesus and Cynomolgus macaques exposed to 5.8 Gy total-body gamma radiation. A chi-square test was performed with SPSS and showed no significant difference between species.

The survival rates for the two groups, two species of macaques, acutely exposed to^60^Co gamma rays to total doses of 6.5 Gy, were 70% and 12.5% for the rhesus and cynomolgus groups, respectively (Fig. 1). The first death for this dose was observed within the rhesus group on day 13, with subsequent deaths occurring through day 17 post-irradiation. By contrast, the first death within the cynomolgus group occurred on day 16, with subsequent deaths continuing through day 28. At the lower level of radiation exposure (5.8 Gy), the overall survival rate within the rhesus group remained at 70%, whereas the survival rate within the cynomolgus group was 25%, a value marginally higher than that noted at the higher dosing level (Fig. 2). The early deaths within the rhesus macaque group resulting from radiation exposures to 5.8 Gy were initially observed at day 15 and continued through day 18. The first death in the cynomolgus group that received this dose was observed on day 17 and continued through day 27 post-irradiation. A Chi-square test was performed to assess significance between rhesus and cynomolgus macaques for both radiation dose groups. Although no significance in survival rates was detected between rhesus and cynomolgus macaques exposed to 5.8 Gy (70% vs. 25%, respectively; *p*-value = 0.058), a significant difference was detected in survival rates of the two-macaque species exposed at the higher dosing level (6.5 Gy) (70% vs. 12.5%, respectively; *p*-value = 0.015). Although the overall survival rates for rhesus macaques were indeed higher than that of the cynomolgus macaques exposed to either of the radiation doses tested (6.5/5.8 Gy), these differences proved (via chi-square analysis) to be statistically significant only at the higher dose (6.5 Gy), but not at the lower dosing level (5.8 Gy). The small sample size contributed, no doubt, to the latter statistical finding. In both doses of radiation, early deaths started occurring in both macaque species at approximately the same time following irradiation; however, subsequent deaths continued to occur in the cynomolgus group, as compared to the rhesus group. In sum, rhesus macaques exposed to 5.8 and 6.5 Gy showed no difference in overall survival in this study. By contrast and under experimental conditions used previously for ‘rhesus macaque’-based studies, a TBI dose of 6.5 Gy would be expected to yield lower survival rate of ~ 50% and not a higher survival rate of ~ 70% as noted here.

Survival outcomes of male rhesus and cynomolgus macaques exposed to 5.8 or 6.5 Gy radiation in relation to age and body weight have been assessed and comparisons made: these data/comparisons indicate that the small differences in the age and body weight of the test animals at the time of exposure had little influence on survival outcomes (Table 1). However, and by contrast, at the lower dosing level of 5.8 Gy, the slightly heavier body weights of the two surviving cynomolgus macaques might have provided a ‘survival benefit’. (Table 1).

**Table 1:** Survival outcomes of male rhesus and cynomolgus macaques exposed to 5.8 or 6.5 Gy ionizing radiation in relation to age and body weight.

Radiation Dose	Species	Survivors	N	Body Weight (kg)				Age (months)				Survival (%)
				Range	Average	SD	*p* value1	Range	Average	SD	*p* value1	
5.8 Gy	Rhesus	Total	10	4.8–9.0	7.1	1.2	0.085	53–72	62.8	6.8	0.486	70
		Survival	7	4.8–9.0	7.4	1.4		53–72	62.9	7.1		
		Deaths	3	6.4–6.7	6.5	0.2		54–67	62.7	7.5		
	Cynomolgus	Total	8	5.5–6.9	6.1	0.6	0.024	58–68	65.0	3.0	0.500	25
		Survival	2	6.5–6.8	6.7	0.2		65	65.0	0		
		Deaths	6	5.5–6.9	6.0	0.6		58–68	65.0	3.6		
6.5 Gy	Rhesus	Total	10	4.3–5.3	4.8	0.4	0.338	43–56	50.9	3.9	0.068	70
		Survival	7	4.3–5.3	4.7	0.4		43–56	49.9	4.1		
		Deaths	3	4.4–5.3	4.9	0.5		52–56	53.3	2.3		
	Cynomolgus	Total	8	5.6–7.5	6.4	0.8	–	56–82	68.3	7.6	–	12.5
		Survival	1	5.9	5.9	–		56	56	–		
		Deaths	7	5.6–7.5	6.4	0.8		63–82	70.0	6.2		

SD, standard deviation.

1p values determined by Student’s t-test: single tailed distribution, two samples, unequal variance.

Nevertheless, and based on the observed survival rates for the two groups, it is apparent that cynomolgus macaques have a greater mortality rate than the rhesus regardless of the radiation dose (Table 1). It is also worth noting that all groups have very small sample sizes, emphasizing the need to conduct such studies in future with larger sample sizes based on availability of resources.

### Blood response patterns

**Fig. 3:**
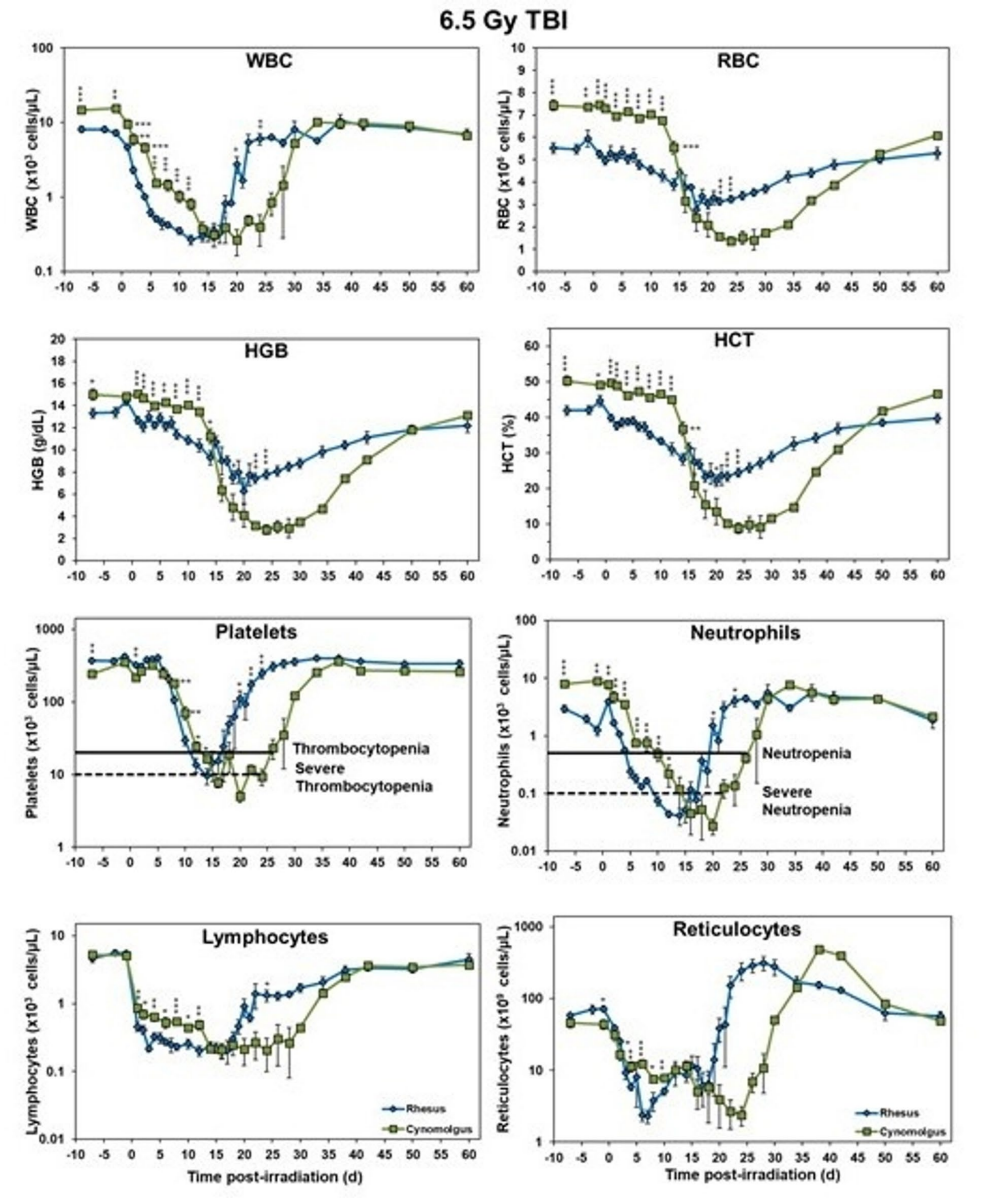
Complete blood counts of male Rhesus and Cynomolgus macaques exposed to 6.5 Gy total-body gamma radiation. The data for each timepoint is presented as the mean for each group. A significant difference between the groups is denoted by the number of asterisks where *, **, and *** correspond to *p* < 0.05, 0.01, and 0.001, respectively. The groups were not statistically compared past d 24 due to a limitation in cynomolgus sample size of less than 3 NHPs.

The effects of acute radiation exposure on the CBCs of the two groups of NHPs (rhesus and cynomolgus macaques) undergoing 6.5 Gy TBI are shown in Fig. 3. Overall, there is a trend where initial values of cynomolgus macaques are higher than rhesus but they consistently drop below the rhesus around d 15 and show prolonged recovery back to pre-irradiation values compared to rhesus where recovery is early. There are a few exceptions which are observed for platelets and reticulocytes as initially the counts are more depressed in cynomolgus macaques and lymphocytes show no initial difference but all parameters quickly follow the same trend. Generally, blood counts of all analyzed parameters showed significant differences on most study days before reaching the nadir.

**Fig. 4:**
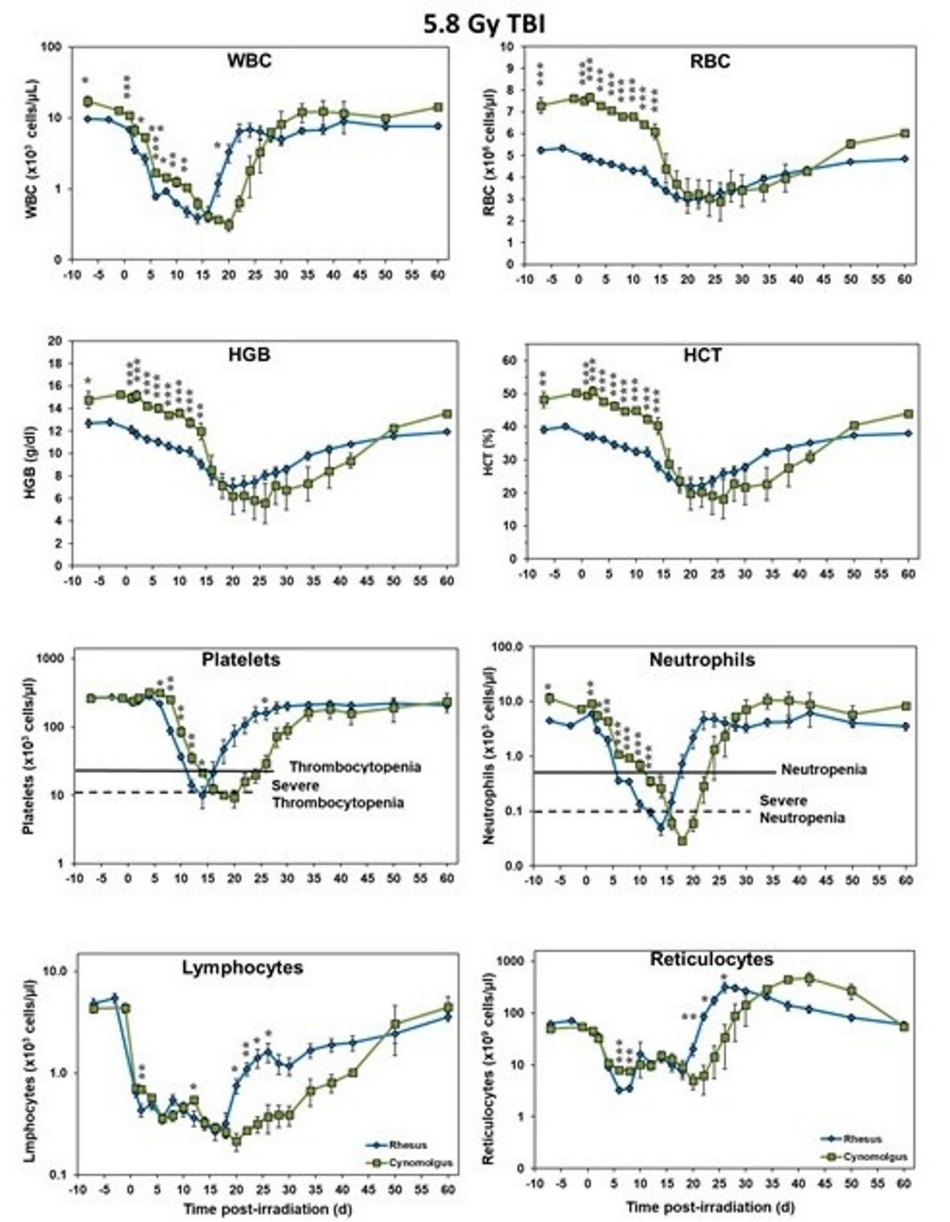
Complete blood counts of male Rhesus and Cynomolgus macaques exposed to 5.8 Gy total-body gamma radiation. The data for each timepoint is presented as the mean for each group. A significant difference between the groups is denoted by the number of asterisks where *, **, and *** correspond to *p* < 0.05, 0.01, and 0.001, respectively. The groups were not statistically compared past d 27 due to a limitation in cynomolgus sample size of less than 3 NHPs.

Similar to the trend noted for the 6.5 Gy, the rhesus group exposed to 5.8 Gy shows lower initial counts for most blood parameters investigated which reached a nadir around d 15 and rose to pre-irradiated values earlier than the cynomolgus group (Fig. 4). Lymphocytes and reticulocytes were in part exceptions as the CBCs for cynomolgus macaques, in contrast to the rhesus macaques, tended to show marginally lower blood cell levels during the pre-exposure period; whereas blood platelet levels were initially comparable for the two species (Fig. 4). In brief, most of the analyzed blood components were significantly different between the two species for a majority of the study time points leading up to the nadir with the exception of lymphocytes and reticulocytes. Additionally, WBC and platelet counts were significantly different on a single day after the nadir. Absolute counts for neutrophils and lymphocytes of all animals have been provided as supplementary Tables 1–4.

### Blood chemistry response

**Fig. 5:**
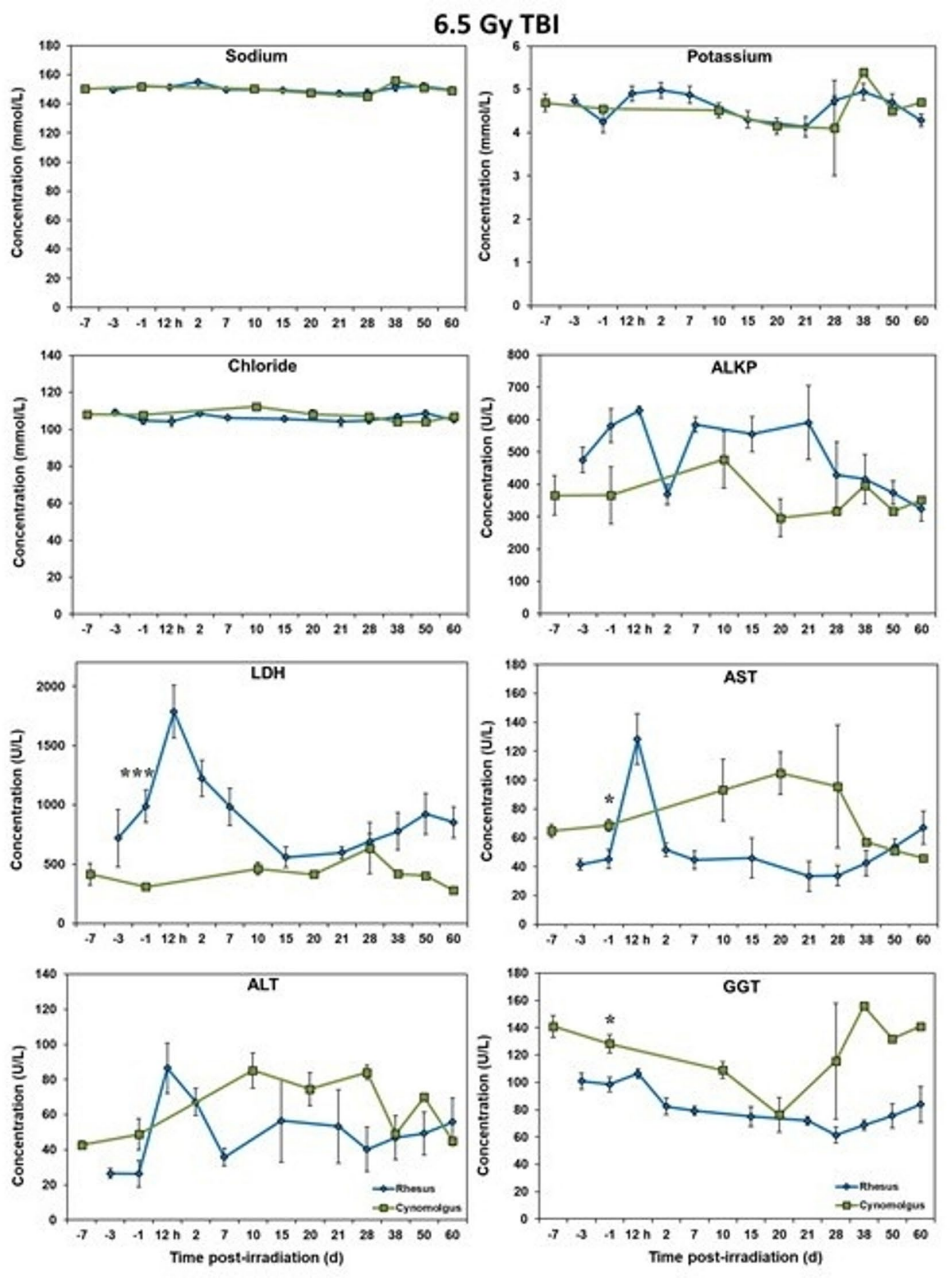
Chemistry analysis results of male Rhesus and Cynomolgus macaques exposed to 6.5 Gy total-body gamma radiation. The data for each timepoint is presented as the mean for each group. A significant difference between the groups is denoted by the number of asterisks where * and *** correspond to *p* < 0.05 and 0.001, respectively. The groups were not statistically compared past d 24 due to a limitation in cynomolgus sample size of less than 3 NHPs.

**Fig. 6:**
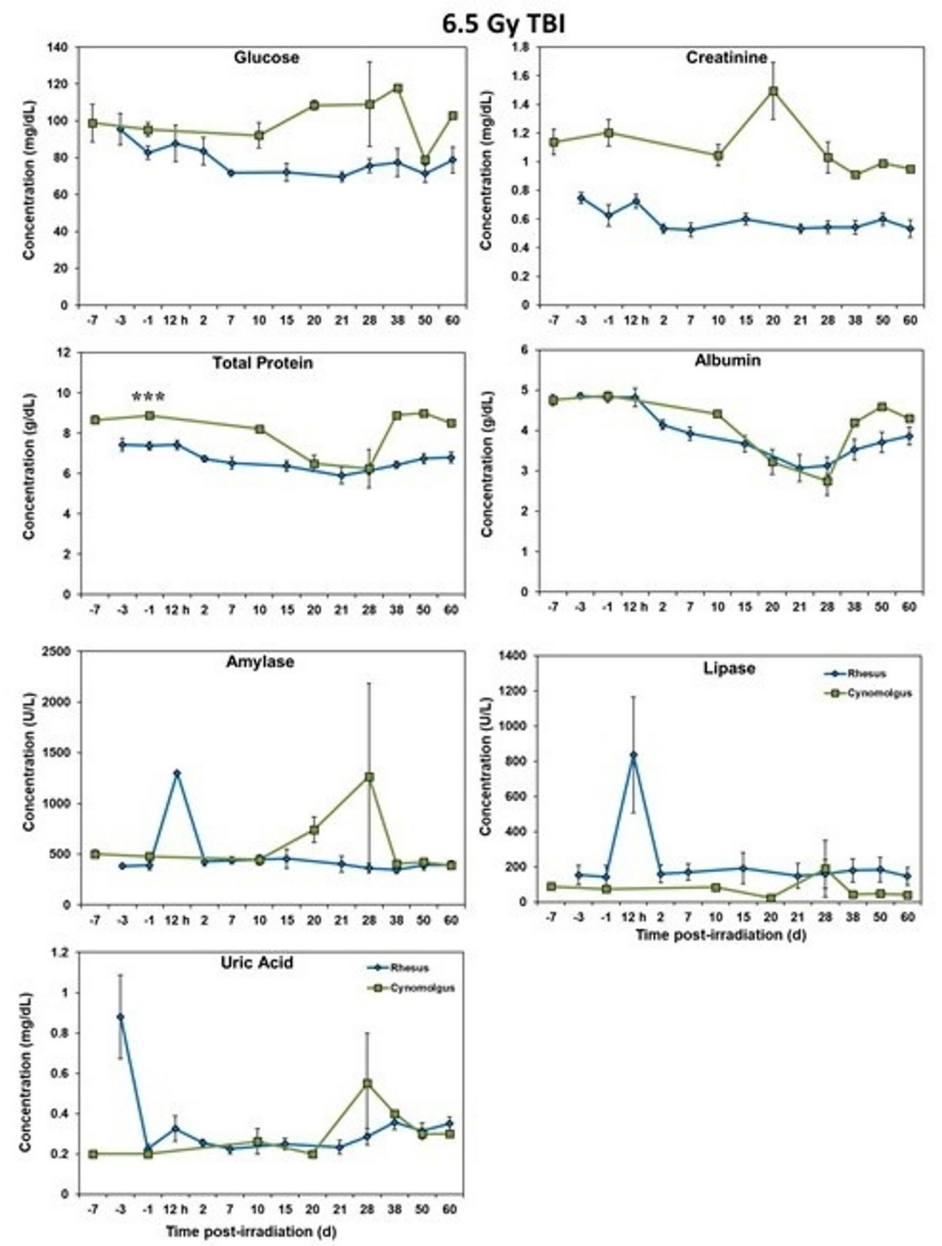
Chemistry analysis results of male Rhesus and Cynomolgus macaques exposed to 6.5 Gy total-body gamma radiation. The data for each timepoint is presented as the mean for each group. A significant difference between the groups is denoted by the number of asterisks where * and ** correspond to *p* < 0.05 and 0.01, respectively. The groups were not statistically compared past d 24 due to a limitation in cynomolgus sample size of less than 3 NHPs.

**Fig. 7:**
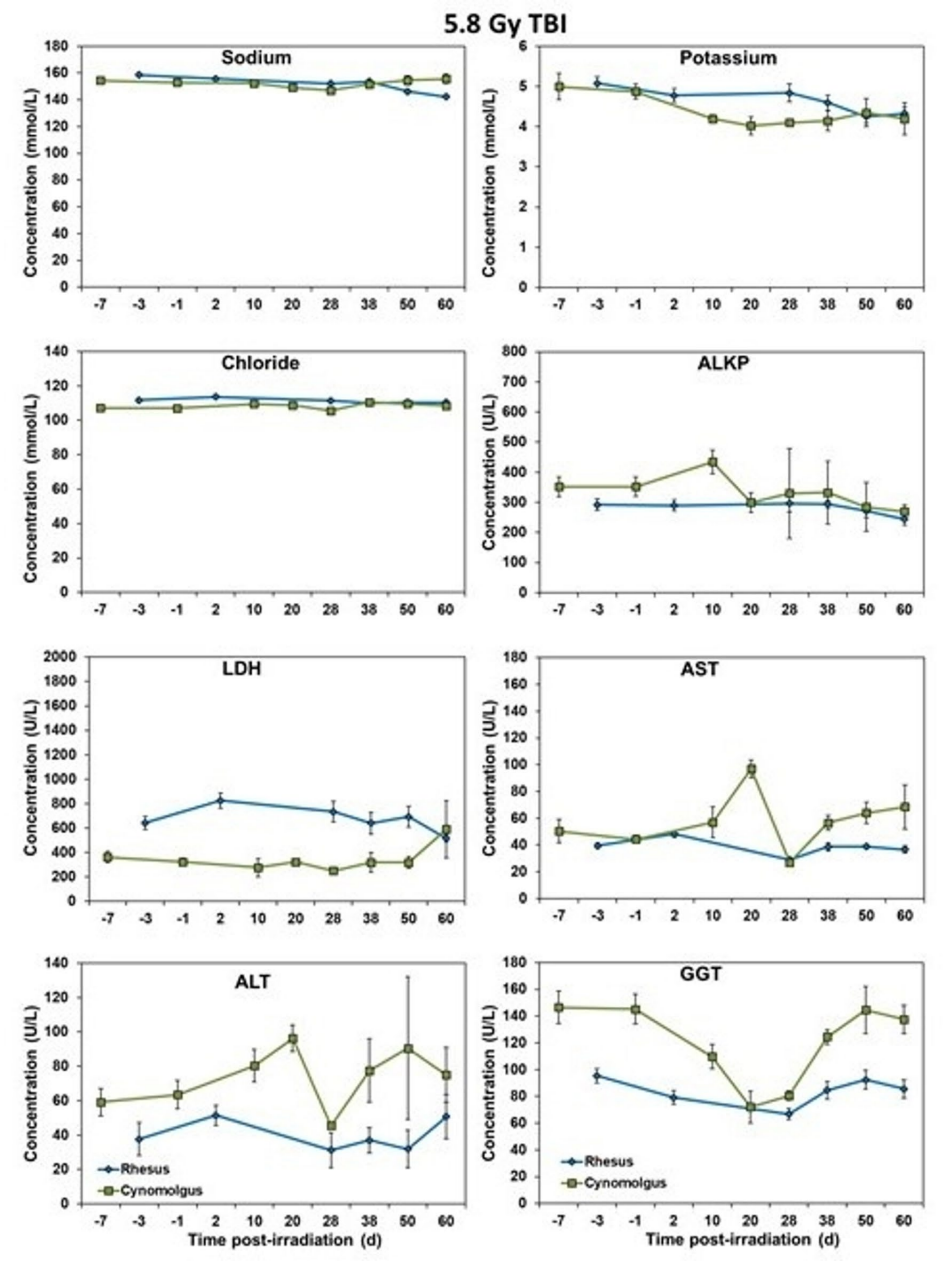
Chemistry analysis results of male Rhesus and Cynomolgus macaques exposed to 5.8 Gy total-body gamma radiation. The data for each timepoint is presented as the mean for each group. No significant differences were noted between the group at any time point.

**Fig. 8:**
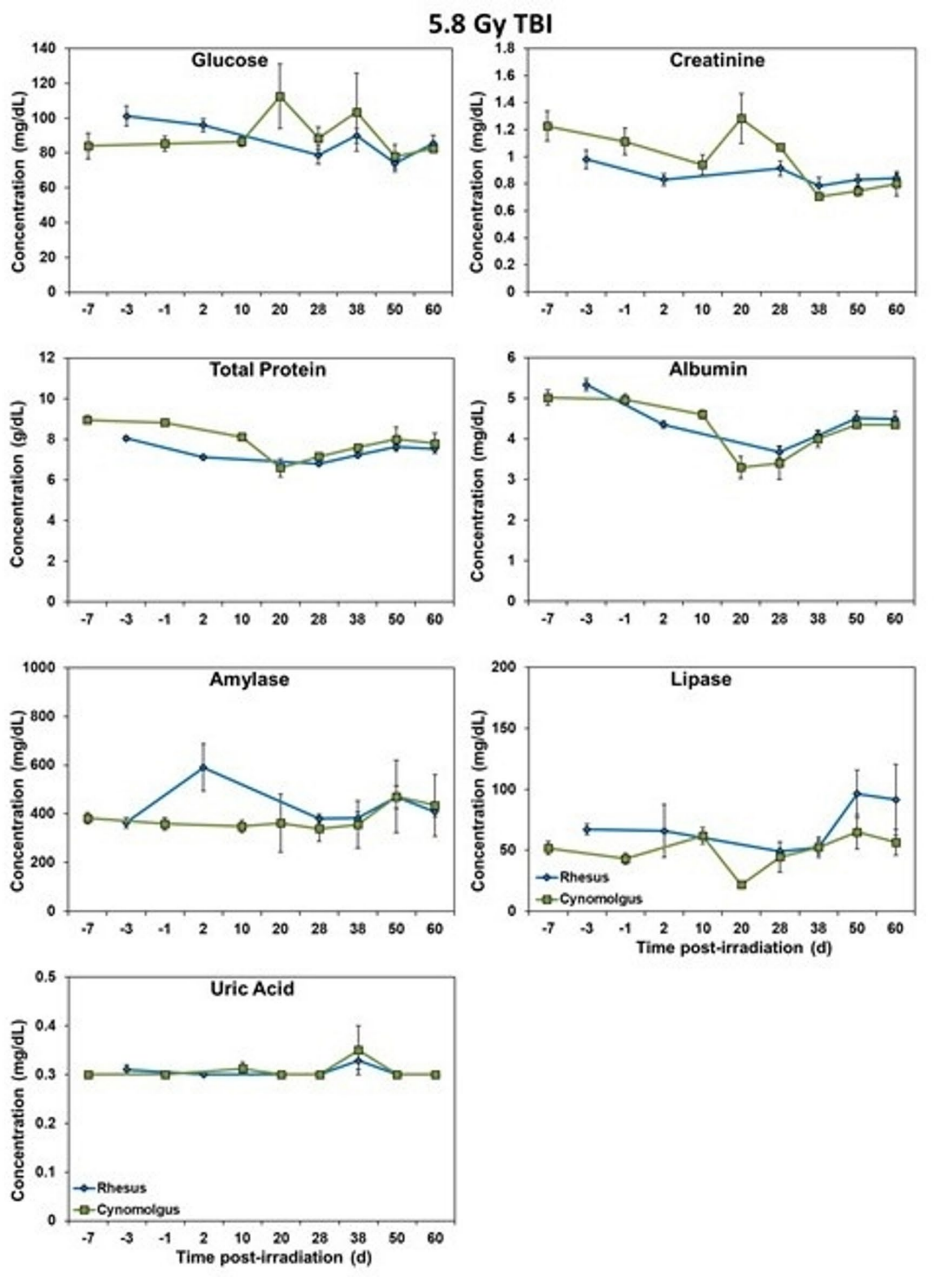
Chemistry analysis results of male Rhesus and Cynomolgus macaques exposed to 5.8 Gy total-body gamma radiation. The data for each timepoint is presented as the mean for each group. No significant differences were noted between the group at any time point.

Blood chemistry analyses of rhesus and cynomolgus macaques were analyzed for NHPs exposed to potentially lethal doses (6.5/5.8 Gy) of total-body gamma radiation and the results are shown in Figs. 5, 6, 7 and 8. Relative to the major blood ions (Na, Cl, K), values were remarkably similar for the two-macaque species over the course of the experiment (Figs. 5 and 7). By contrast, the two-macaque species were remarkably inconsistent and often quite different relative to the other components of the measured blood chemistries; e.g., the measured blood enzymes, AST, ALT, GGT, ALKP, etc. (Figs. 6 and 8).

### Cytokine response patterns

The measured cytokine response patterns of the two-macaque species appear markedly different (Supplementary Figs. 1 and 2). For example, the responses of selected cytokines (e.g., IL6, IL8, IL10, IL-1β) with rhesus macaques tend to be initially upregulated (4 h) shortly following irradiation (Supplementary Figs. 1 and 2); whereas the cynomolgus macaques tended to exhibit peak cytokine responses much later following exposure (Supplementary Figs. 1 and 2; 20–36 d post-exposure).

## Discussion

Comparing select responses (e.g., survival, hematopoietic, etc.) of two closely related species of NHPs, namely rhesus and cynomolgus macaques, to acute, whole-body, potentially lethal IR, marked differences were noted in this study. There is little doubt that these two species, often used by investigators as the primary large animal model of choice under FDA’s ‘Animal Rule’, are responding differently to these acute IR exposures. Do these noted differences actually matter in terms of assessing the nature and consequences of IR-associated injury, along with evaluating the utility/efficacy of newly discovered MCMs?

For the development and approval of new MCMs for radiation exposure following the US FDA Animal Rule for human use, promising agents need to be investigated in appropriate large animal models for both for efficacy, safety and toxicity, as well as for the identification and utility of select biomarkers. Due to the ethical limitations of evaluating the efficacy of any given MCM in healthy volunteers, the FDA developed a new ‘Animal Rule’, a rule that allows for testing of potentially useful, injury-countering agents (i.e., agents designed to counter the toxic effects of unwanted biological, chemical or radiological/nuclear exposures) in well-characterized animal models^12,50–52^. Further, this Animal Rule and its various testing requirements is readily understood by investigators focusing on MCM identification and development. It is also well recognized and generally accepted by investigators that the agency’s Animal Rule recommendation requires that not only should large animal models be used (along with complementing small animal models), but that they (the models) should be well-defined, and mechanism of injury well-understood. These limitations create a series of logistical challenges for researchers and for corporate pharmaceutical alike partners in trying to move new MCMs through the various research and development steps to get regulatory approval prior to their use in the clinical setting. The limited availability, high procurement cost, and maintenance of a required number of large animals for proper MCM testing highlight the cost of such drug development endeavors.

For the last several years, the supply of rhesus macaques has been under constraint and we, the MCM developers, have few options other than to identify, characterize, and subsequently utilize other large animal models. Cynomolgus macaques have been used in the past for MCM development, although this large animal model is not as well characterized as the rhesus macaque-based model, it clearly needs to be further characterized^53^. In this regard, we have compared the data generated previously from several large animal, macaque-based radiation studies. These studies utilized only a limited number of rhesus and cynomolgus macaques, but they were carried out under nearly identical conditions. As noted here, there are clear and unambiguous differences displayed by these two-macaque species in response to acute irradiation. Most notably, these differences include basic survival patterns and outcomes, as well as patterns of suppression and recovery of blood elements. As the current study seeks to propose the cynomolgus macaque as a possible alternative large animal model to the well-accepted rhesus macaque model for regulatory approval of MCMs, these noted differences (between the two species of macaques) might prove problematic for both researchers and regulators alike. For example, the noted response differences beg a series of important questions as to ‘Which of these two-macaque species best reflects responses of comparably exposed humans? ‘Are there alternative large animal models to be seriously considered and accepted as being valid? ‘Is the rhesus model truly the best available, self-sufficient large animal model? In our view, such questions are both valid and legitimate.

Nevertheless, although the responses might not be exactly the same, they are indeed comparable. Blood chemistry analysis shows little difference between these two species for either radiation dose. CBC recovery patterns were different at various time points but overall followed a similar trend in both species for both doses. Using rhesus macaques, thrombocytopenic severity and duration have been shown to be an important predictor of mortality^54^. A greater mortality rates were observed in cynomolgus macaques who experienced longer durations of severe thrombocytopenia and marked anemia noted by decreased hematocrit values. Cytokine response patterns also appeared at first glance to be markedly different for the two macaque species, especially in terms of the temporal expression patterns, but these differences proved largely to be insignificant statistically due to small sample size. Finally, in terms of overall radiosensitivity as assessed by the 60-day survival metric, there were apparent differences, cynomolgus macaques being somewhat more radiosensitive than the rhesus macaques.

As intriguing as these comparisons may be, we need to note that the study’s two main limitations are (1) the relatively small sample size of the animal groups (n = 10 or 8/group) and (2) only a single sex of animals (males) were used (i.e., due to the unavailability of female cynomolgus macaques at the time of animal procurement). A further note or two on these limitations is warranted: First, with time, supply situation of cynomolgus is improving; second, we have to date, separately conducted rhesus studies with both males and females under comparable experimental conditions; and third, appropriate comparisons using animals of both sexes will be made in the not-to-distant future depending on availability of female cynomolgus and funding for such studies. It also needs to be pointed out that the current study only compares rhesus and cynomolgus macaques at only two TBI doses, 6.5 and 5.8 Gy. Clearly, comparing rhesus and cynomolgus exposed to broader range of radiation doses would not only be interesting, but essential in order to make the case for using cynomolgus macaques as an alternative large animal model for radiation injury. Similarly, partial-body exposure and various levels of supportive care use need to be compared using both macaques under identical experimental conditions.

In the final analysis, we agree that there were limitations in this study; particularly the use of a limited number and only male animals and two radiation doses used. We plan to conduct additional studies in the future to overcome these limitations as soon as we secure additional funding. Without question, the findings of this study are interesting and support the basic concept that both macaques of the large animal species can be used for advanced development of MCMs. This most certainly includes the evaluation of both the efficacy and the safety of various MCMs under development.

## Electronic supplementary material

Below is the link to the electronic supplementary material.

Supplementary Material 1

Supplementary Material 2

Supplementary Material 3

## Data Availability

Data is provided within the manuscript or supplementary information files.
