# Supplementary figures and images for: Comparison of sensitivity of rhesus and cynomolgus macaque for acute radiation effects
Source: Sci Rep. 2025 Jul 8;15:24417. doi: 10.1038/s41598-025-10094-y (PMC12238281; doi:10.1038/s41598-025-10094-y)

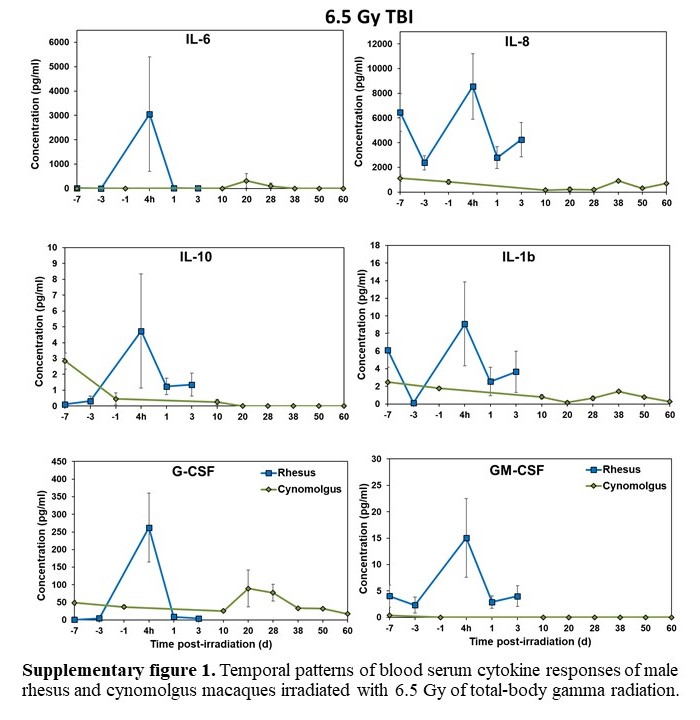

Supplement: Supplementary file 1 — Supplementary Material 1 [file 41598_2025_10094_MOESM1_ESM.jpg]

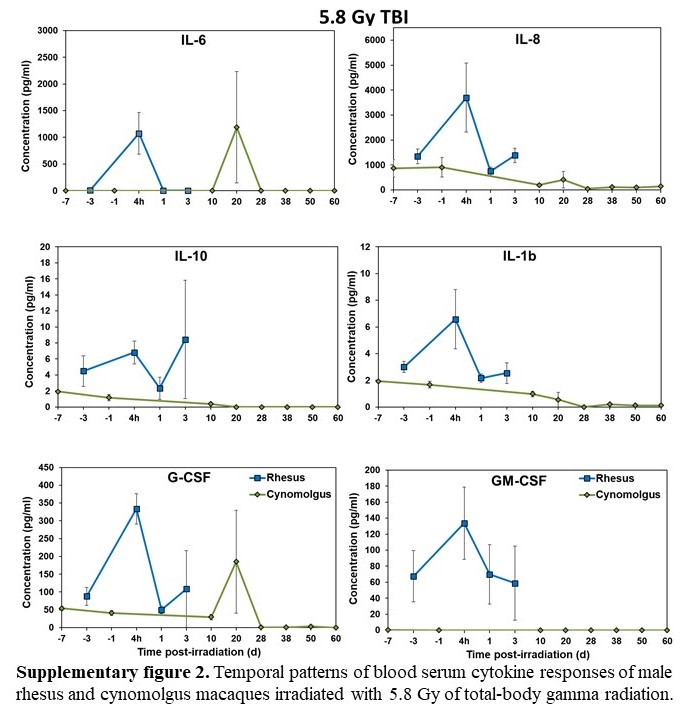

Supplement: Supplementary file 2 — Supplementary Material 2 [file 41598_2025_10094_MOESM2_ESM.jpg]
